# The Pyrolytic Profile of Lyophilized and Deep-Frozen Compact Part of the Human Bone

**DOI:** 10.1100/2012/162406

**Published:** 2012-04-24

**Authors:** Jolanta Lodowska, Daniel Wolny, Sławomir Kurkiewicz, Ludmiła Węglarz

**Affiliations:** ^1^Department of Biochemistry, Medical University of Silesia, Narcyzow 1, 41-200 Sosnowiec, Poland; ^2^Department of Biopharmacy, Medical University of Silesia, Narcyzow 1, 41-200 Sosnowiec, Poland; ^3^Department of Instrumental Analysis, Medical University of Silesia, Narcyzow 1, 41-200 Sosnowiec, Poland

## Abstract

*Background*. Bone grafts are used in the treatment of nonunion of fractures, bone tumors and in arthroplasty. Tissues preserved by lyophilization or deep freezing are used as implants nowadays. Lyophilized grafts are utilized in the therapy of birth defects and bone benign tumors, while deep-frozen ones are applied in orthopedics. The aim of the study was to compare the pyrolytic pattern, as an indirect means of the analysis of organic composition of deep-frozen and lyophilized compact part of the human bone. *Methods*. Samples of preserved bone tissue were subjected to thermolysis and tetrahydroammonium-hydroxide- (TMAH-) associated thermochemolysis coupled with gas chromatography and mass spectrometry (Py-GC/MS). *Results*. Derivatives of benzene, pyridine, pyrrole, phenol, sulfur compounds, nitriles, saturated and unsaturated aliphatic hydrocarbons, and fatty acids (C12–C20) were identified in the pyrolytic pattern. The pyrolyzates were the most abundant in derivatives of pyrrole and nitriles originated from proteins. The predominant product in pyrolytic pattern of the investigated bone was pyrrolo[1,2-**α**]piperazine-3,6-dione derived from collagen. The content of this compound significantly differentiated the lyophilized graft from the deep-frozen one. Oleic and palmitic acid were predominant among fatty acids of the investigated samples. The deep-frozen implants were characterized by higher percentage of long-chain fatty acids than lyophilized grafts.

## 1. Introduction

Historically, the first bone graft was implanted to a patient suffering from inflammation of humerus shaft over 120 years ago [1, 2]. At present, bone transplantation is used in the treatment of bone birth defects, nonunion of bone after the injury, bone necrosis, inflammatory lesions, arthrosis, scoliosis, hip dysplasia (hypoplasia), and after the resection of bone benign tumors [3]. Bone grafts, besides serving as a structural support, should also induce osteogenesis in a recipient tissue. Stimulation of this process depends on bone morphogenetic proteins that are bound with collagen. The bone grafts are considered as the best grafting material because of their osteogenetic, osteoconductive, and osteoinductive properties. However, their immunogenicity may lead to the graft rejection. Deep freezing and lyophilization are used to overcome this problem [4]. Both biological properties (immunogenicity, time of resorption, and osteoinduction) and mechanical strength of allogenic grafts depends on their chemical properties that are correlated with the formation of free radicals and cleavage and cross-linking of collagen, that, in turn, depend on both the procedure of conservation (deep-freezing, freeze-drying) and conditions of radiation sterilization (dose and temperature) [5].

As early as at the beginning of 20th century, the first studies on methods of bone grafts storage were initiated. Albie used low temperature to preserve bone, and, on the contrary, Gollie employed high temperature for this purpose [4]. The drying of implants in autoclave at 120°C was proposed by Rittner. The freezing of grafts in body fluids such as blood or serum or their storage in mineral oil, alcohol, formol, or ether was recommended by some researchers [6–9]. In 1951, Kreuza et al. proposed to lyophilize the bone grafts, and, in 1956, Turner introduced a method of bone implants storage consisting in freeze-drying of radiation-sterilized tissue [4].

The aim of this study was to compare the pyrolytic pattern, as an indirect means of the analysis of the organic composition of compact part of the bone preserved by a deep-freezing and lyophilization.

## 2. Experimental

### 2.1. Material

Compact parts of thigh bones derived from human corpses aged 45–60 years, which have been assigned to a purpose of further grafting, were used in the study. Bone compact parts have been obtained from the tissue collection of the Center of Blood Donation in Katowice, and they were milled into fine powder by a freezer mill in order to homogenize and improve the efficiency of their further processing.

### 2.2. Equipment and Procedure

Samples of the human thigh-bone compact structure were pyrolysed at 660°C, and the obtained products of thermolysis and 10% methanolic tetramethylammonium hydroxide-induced thermochemolysis (TMAH) were analyzed by GC/MS with the use of the Py-GC/MS system. The Curie-Point Pyrolyser 795050 (Pye-Unicam) was directly attached to a capillary column HP5-MS (60 m × 0.32 mm × 0.5 *μ*m) of a Hewlett Packard HP-5890 gas chromatograph (GC) series II, coupled with a Hewlett Packard HP-5989A mass spectrometer. For experimental data collection and mass spectra interpretation, the Chemstation software G1034C ver. C.02.00 (Hewlett Packard) was used.

Helium was used as a carrier gas at a constant pressure of 15 psi. Thermolysis and thermochemolysis were conducted in tubular ferromagnetic wire inductively heated to Tc of 610°C by a pyrolytic unit. The temperature of the pyrolyser oven was 220°C, and the samples were heated for 5 s. The initial temperature of the GC oven was set to 40°C for 5 min, then increased to 250°C at 10°C/min, maintained for 16 min, and increased again at 10°C/min to a final temperature of 270°C held for 10 min. To neglect the solvent (TMAH-) derived peak, the analysis was recorded after 5 min. The temperature of ion source in spectrometer was maintained at 200°C and that of a quadruple at 100°C. All spectra were collected using 70 eV electron ionization. Mass spectra from 33 to 500 m/z were accumulated, and peaks were assigned by comparison with the library data of the 7th Issue Wiley Library.

### 2.3. Statistics

*T*-test for independent samples was used to compare the products of thermolysis and thermochemolysis of lyophilized and deep-frozen bone tissue samples. Normality of distributions has been verified by the Shapiro-Wilk test, while the homogeneity of variances has been ascertained by the *F*-test or the Levene test. The results have been considered significant at *P* < 0.05. Numerical cluster analysis was used to determine the similarity of fatty acid profiles of investigated tissues. A dendrogram based on the Gammas correlation coefficient as the distance measure was generated. Statistical analysis was performed using Statistica 8.0 software.

## 3. Results

**Figure 1:**
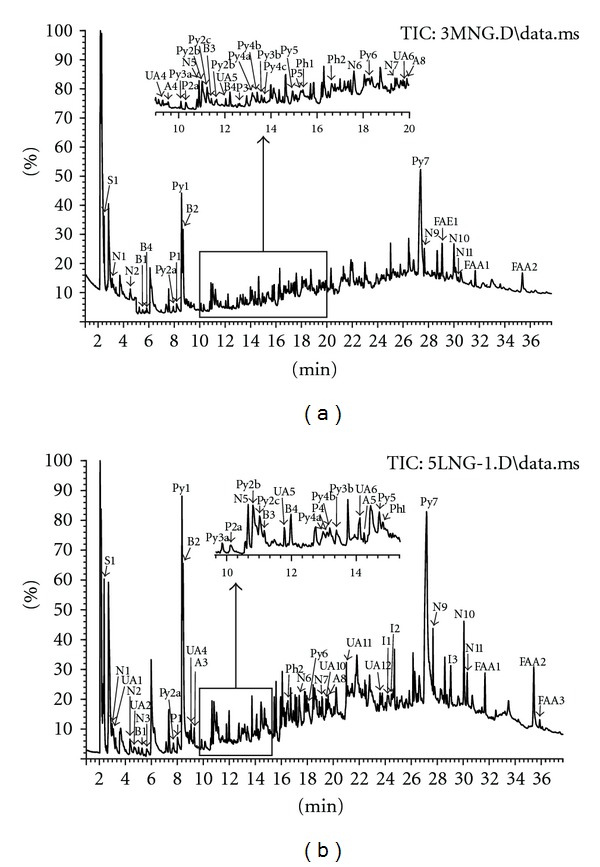
Chromatograms of thermolysis products of deep-frozen (a) and lyophilized (b) bone tissue.

**Figure 2:**
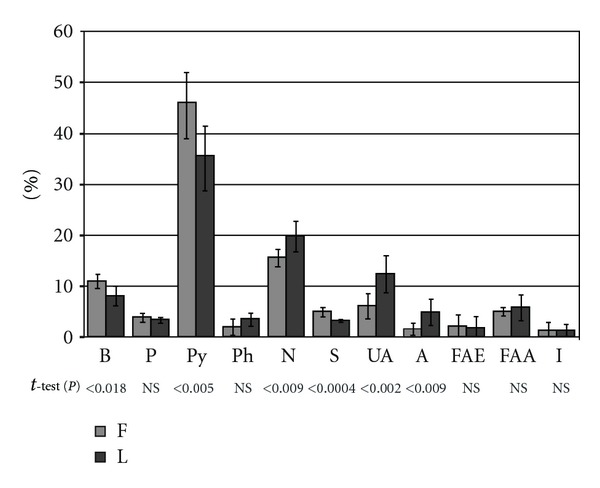
Quantitative relations between the main groups of thermolysis products derived from the components of the compact part of human bone preserved by deep-freezing (F) or lyophilization (L) and classified according to their chemical structure.

Chromatograms obtained after the thermolysis of deep-frozen and lyophilized bone are presented in Figure 1. Identified analytes, classified according to their chemical structure as derivatives of benzene, pyridine, pyrrole, phenol, sulfur compounds, nitriles, saturated and unsaturated aliphatic carbohydrates, and the derivatives of fatty acids, are shown in Table 1. It was found that the derivatives of pyrrole were predominant in a pyrolytic pattern of both analyzed kinds of biomaterials, but, in the deep-frozen bone, the content of these compounds was greater. It was mainly caused by the diverse quantity of pyrrolo[1,2-*α*]piperazine-3,6-dione, the amount of which in the lyophilized tissue was lower by 9.4% compared to the deep-frozen one (Table 1, Figure 2).

**Figure 3:**
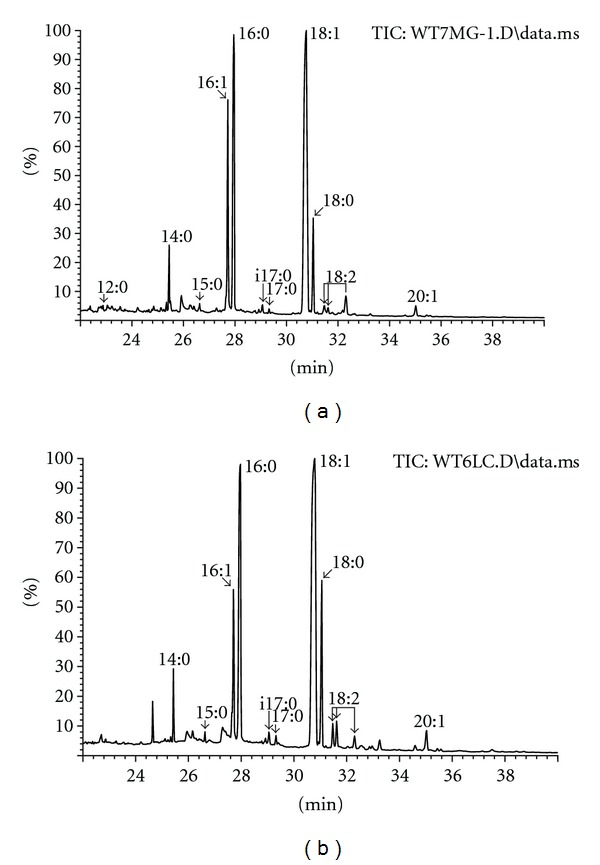
Chromatograms of fatty acid derivatives formed during the thermolysis in the presence of TMAH of deep-frozen (a) and lyophilized (b) bone.

**Figure 4:**
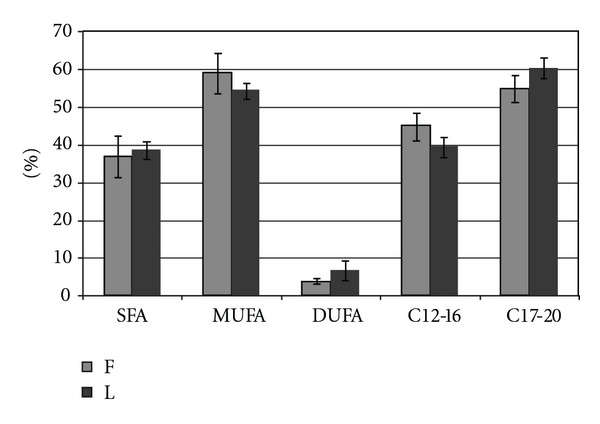
Comparison of the carbohydrate chain length and degree of saturation of fatty acids, the components of deep-frozen (F) and lyophilized (L) compact part of the human bone (SFA: saturated fatty acids; MUSFA: monounsaturated fatty acids; DUSFA: diunsaturated fatty acids; C12–16: fatty acids with chain length of 12 to 16 carbons; C17–20: fatty acids with chain length of 17 to 20 carbons).

The results of chromatographic analysis of fatty acids derivatives obtained by thermochemolysis in the presence of TMAH of lyophilized and deep-frozen bone tissue are shown in Figure 3. The comparison of fatty acid methyl esters profile (Figures 3 and 4, Table 2) revealed higher percentage of long chain fatty acids (C17–C20) in deep-frozen grafts than in lyophilized ones. The statistically significant differences in mean percentage of C12–C16 (*P* = 0.0054) and C17–C20 (*P* = 0.0054) fatty acids between analyzed biomaterial were found. The diverse content of mono- and diunsaturated fatty acids (*P* = 0.0459 and *P* = 0.003, resp.) was also observed.

**Figure 5:**
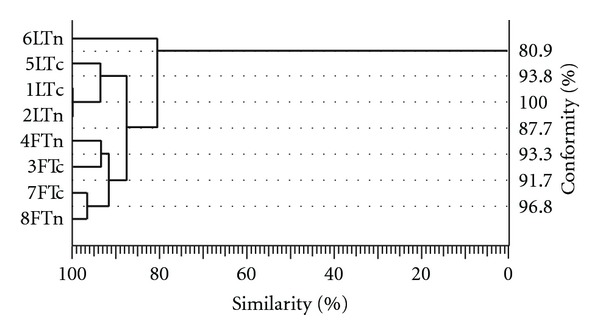
Dendrogram of fatty acid profiles similarity created by a numerical cluster analysis (F: deep-frozen; L: lyophilized; Tn: thin; Tc: thick).

Numerical cluster analysis (Figure 5) showed over 80% similarity of bone grafts fatty acid pattern. Based on the fatty acid profile, the lyophilized and deep-frozen bone implants were classified to separate homogenous clusters; therefore, the resulting fatty acid profile is dependent on the method of grafts preservation. The similarity of lyophilized and deep-frozen bone tissue was at 87.7%.

## 4. Discussion

Both massive bone allografts and morselized bone pieces, as filling material, are used in orthopedic surgery [10, 11]. Bone grafts are required in about 15% of all reconstructive operations. They are preserved by deep-freezing and lyophilization. The process of deep-freezing affects not only immunogenicity of the grafts but also their physical and especially mechanical properties [12–15]. The investigation of Pelker et al. [14] proved that deep-freezing of bone implants at −196°C increased their mechanical strength by about 10%, whereas, according to Komender [12], this property was not affected when the process was conducted at −78°C. The compressive strength of grafts was increased by 20% by lyophilization followed by freezing [14]. The storage of bone implant at −20°C does not affect its physical properties [16], but it does not prevent the enzymatic degradation of its components.

The derivatives of benzene, pyridine, pyrrole, phenol, sulfur compounds, nitriles, saturated and unsaturated aliphatic hydrocarbons, and fatty acids (C12–C20) were identified in the pyrolytic pattern of bone implants. The large amount of nitrogen-containing compounds (derivatives of pyrrole, pyridine, and nitriles) confirms the significant protein content in the investigated material [17]. Both Gleixner et al. [18] and Poinar and Stankiewicz [19] claim that this kind of compounds derive from proteins. Predominant among not only pyrrole derivatives but also among all analytes pyrrolo[1,2-*α*]piperazine-3,6-dione was formed from dipeptide sequences containing proline. This diketopiperazine derivative is a product of reaction of imine group of N-terminal proline with amide-bound carboxylic group of adjacent glycine [20], which occurs in characteristic for collagens Pro-Gly sequences [21]. Toluene, ethylbenzene, benzene, and nitriles (benzylnitrile and benzenepropanenitrile) originated from phenylalanyl residues as a result of thermal degradation of the organic constituents of bone [22]. Phenylalanine may be transformed to styrene, whereas tyrosine forms styrene and phenol during thermolysis [23]. These derivatives are found in smaller amounts as compared to other products of thermolysis because the content of their parent amino acids is small in collagen—the main component of bone [24, 25]. Tyrosine is a constituent of bone sialoprotein (BSP) that accounts for 10 to 15% of noncollagenous proteins of bone [26]. The scant amount of sulfur-containing products of thermolysis results from small quantity of sulfur-containing amino acids in bone collagen.

Among fatty acids of bone only oleic and palmitic acid derivatives were identified by thermolysis coupled with GC/MS. These compounds are little volatile that significantly hinders their determination by the gas chromatography method. Therefore, thermolysis assisted by methylation in the presence of methanolic solution of tetramethylammonium hydroxide (TMAH) was used. Interpretation of the data obtained by this method proved the presence of fatty acids with chain length of 12 to 20 carbons in analyzed tissue. As in case of thermolysis without TMAH, oleic and palmitic fatty acids were predominant among identified fatty acid derivatives. A particularly large amount of these fatty acids in compact part of bone was also observed by Dołęgowska et al. [27] and Kagawa et al. [28]. Comparing fatty acid profiles of deep-frozen and lyophilized bone graft, it was observed that deep-frozen bone was abundant in long-chain fatty acids. This can be caused by the loss of more volatile short-chain fatty acids during the process of lyophilization. The pyrolytic pattern of deep-frozen grafts was richer in pyrrolo[1,2-*α*]piperazine-3,6-dione, that is probably associated with a larger quantity of collagen in this kind of tissue. Płomiński and Kwiatkowski [4] reported that, due to the inhibition of protein degrading enzymes at −70°C, the quantity of proteins was higher in the deep-frozen tissue than in lyophilized one. In deep-frozen bone grafts, radiation-induced degradation of collagen is significantly reduced, thus their mechanical strength is greater and resorption *in vivo* is slower than in the lyophilized tissue [5]. In 1960s, it was noticed that changes in the properties of collagen were associated with a hydratation of biomaterial. The properties of collagen more intensively alter during the irradiation of dehydrated collagen (lyophilized graft), because cleavage of the collagen polypeptide chains occurs, what significantly increases *in vivo* solubility of this protein and decreases its mechanical strength. On the other hand, radiation sterilization of the hydrated tissue (deep-frozen graft and fresh tissue) causes radiolysis of water and formation of free radicals which in turn cross-link the collagen chains [5], and it is commonly known that degree of collagen cross-linking influences its physicochemical properties.

## 5. Conclusion

The percentage of pyrrolo[1,2-*α*]piperazine-3,6-dione, thermolytic marker of collagen, is greater in case of pyrolysis of deep-frozen graft as compared to lyophilized one. On the basis of fatty acid profile, the deep-frozen and lyophilized tissues can be classified to separate homogenous clusters. Although fatty acid profiles of these tissues are qualitatively similar, they differ in the quantity of particular analytes. The content of short-chain fatty acids is smaller in the lyophilized grafts as compared to deep-frozen ones, which can be a consequence of the method used for their preservation.

## Figures and Tables

**Table 1:** The quantity of particular thermolysis products derived from the components of compact part of the human bone preserved by deep-freezing (F) or lyophilization (L) and used for transplantation, expressed as percentage of a given analyte in a total identified compounds.

Symbol of compounds group	Symbol of compound	Thermolysis product	%AUP ± SD	
			F	L
B	B1	Benzene	0.40 ± 0.10	0.33 ± 0.08
	B2	Toluene	9.33 ± 1.39	6.53 ± 1.70
	B3	Ethylbenzene	0.54 ± 0.24	0.43 ± 0.30
	B4	Styrene	0.73 ± 0.25	0.86 ± 0.33

P	P1	Pyridine	1.33 ± 0.22	1.11 ± 0.19
	P2a	2-Methylpyridine	1.09 ± 0.51	0.95 ± 0.23
	P2b	3-Methylpyridine		
	P3	2-Ethylpyridine	0.24 ± 0.47	0.11 ± 0.13
	P4	Dimethylpyridine	0.26 ± 0.35	0.41 ± 0.24
	P5	Pyridinamine	0.95 ± 0.38	0.85 ± 0.57

Py	Py1	Pyrrole	7.31 ± 1.25	6.19 ± 1.52
	Py2a	1-Methylpyrrole		
	Py2b	2-Methylpyrrole	5.52 ± 1.16	4.52 ± 0.69
	Py2c	3-Methylpyrrole		
	Py3a	1-Ethylpyrrole	0.48 ± 0.43	0.77 ± 0.42
	Py3b	2-Ethylpyrrole		
	Py4a	2,3-Dimethylpyrrole		
	Py4b	2,5-Dimethylpyrrole	1.81 ± 0.96	1.76 ± 0.29
	Py4c	2,4-Dimethylpyrrole		
	Py5	2-Ethyl-4-methylpyrrole	0.30 ± 0.35	0.38 ± 0.61
	Py6	Pyrrole-2-carbonitrile	0.46 ± 0.92	1.26 ± 1.52
	Py7	Pyrrolo-[1,2-*α*]-piperazine-3,6-dione	30.13 ± 5.57	20.71 ± 2.22

Ph	Ph1	Phenol	0.57 ± 0.72	1.22 ± 0.72
	Ph2	4-Methylphenol	1.46 ± 1.03	2.28 ± 0.73

N	N1	2-Propenenitrile	1.73 ± 0.14	1.35 ± 0.25
	N2	Isobutyronitrile	2.90 ± 1.73	3.64 ± 3.54
	N3	Butenenitrile	0.12 ± 0.24	0.44 ± 0.25
	N4	Butanenitrile	0.21 ± 0.25	0.55 ± 0.39
	N5	4-Methyl-pentanenitrile	1.27 ± 0.09	0.92 ± 0.16
	N6	Benzyl nitrile	2.82 ± 0.56	2.81 ± 0.41
	N7	Benzenepropanenitrile	0.96 ± 0.67	1.19 ± 0.73
	N8	Pentadecanenitrile	0.63 ± 1.27	0.50 ± 1.00
	N9	Hexadecanenitrile	1.94 ± 1.91	2.67 ± 1.98
	N10	Octadecenenitrile	2.20 ± 2.57	4.11 ± 1.43
	N11	Octadecanenitrile	0.92 ± 1.47	1.61 ± 0.32

S	S1	Methanethiol	5.01 ± 0.85	3.21 ± 0.28

UA	UA1	3-Penten-1-yne	0.00	0.61 ± 0.71
	UA2	1,3-Cyclohexadiene	0.00	0.29 ± 0.37
	UA3	1-Heptene	0.00	0.39 ± 0.78
	UA4	1-Octene	0.93 ± 0.16	1.04 ± 0.07
	UA5	1-Nonene	0.53 ± 0.10	0.63 ± 0.04
	UA6	1-Decene	0.26 ± 0.52	0.38 ± 0.59
	UA7	1-Undecene	0.81 ± 0.61	0.78 ± 0.17
	UA8	1-Dodecene	0.66 ± 0.78	0.62 ± 0.64
	UA9	Cyclododecene	0.00	1.49 ± 1.20
	UA10	1-Tridecene	0.49 ± 0.57	1.10 ± 1.12
	UA11	1-Tetradecene	1.77 ± 0.56	1.89 ± 0.30
	UA12	1,13-Tetradecadiene	0.17 ± 0.34	0.89 ± 0.90
	UA13	1-Pentadecene	0.00	0.53 ± 0.62
	UA14	1-Heksadecene	0.00	0.47 ± 0.41
	UA15	1-Heptadecene	0.22 ± 0.44	0.13 ± 0.26s
	UA16	1-Octadecene	0.26 ± 0.51	1.16 ± 1.41

A	A1	Hexane	0.00	0.26 ± 0.52
	A2	Heptane	0.00	0.20 ± 0.40
	A3	Octane	0.66 ± 0.49	0.85 ± 0.14
	A4	Nonane	0.13 ± 0.26	0.19 ± 0.28
	A5	Decane	0.04 ± 0.08	0.42 ± 0.05
	A6	Undecane	0.35 ± 0.41	0.36 ± 0.41
	A7	Dodecane	0.00	0.31 ± 0.46
	A8	Tridecane	0.29 ± 0.37	0.86 ± 0.55
	A9	Tetradecane	0.00	0.96 ± 0.95
	A10	Pentadecane	0.15 ± 0.30	0.53 ± 0.40

FAE	FAE1	Isopropyl palmitate	2.17 ± 2.33	1.81 ± 2.35

FAA	FAA1	Hexadecanamide	1.52 ± 0.23	1.74 ± 0.64
	FAA2	9-Octadecenamide	3.37 ± 0.81	3.69 ± 1.77
	FAA3	Octadecanamide	0.21 ± 0.25	0.36 ± 0.10

I	I1	2-Methylpyrazine-5-carboxylic acid	0.00	0.09 ± 0.17
	I2	N-Methyl-7-azabicyclo(2,2,1)hept-2-ene	0.39 ± 0.46	0.24 ± 0.30
	I3	Cyclo(L-prolyl-L-prolyl)	1.00 ± 1.25	0.84 ± 1.08
	I4	Squalene	0.00	0.22 ± 0.26

%AUP ± SD: the percent content of the chromatogram peak area of the compound with reference to the sum of the areas of the peaks of identified analytes ± standard deviation.

**Table 2:** The percentage of fatty acids present in deep-frozen (F) and lyophilized (L) human bone tissue.

FAME	F	L
12 : 0	0.34 ± 0.43	0.00
14 : 0	4.84 ± 1.41	4.09 ± 1.16
15 : 0	1.19 ± 1.00	1.38 ± 0.50
16 : 1	12.59 ± 2.80	8.78 ± 2.35
16 : 0	26.11 ± 4.73	25.30 ± 4.27
*i*17:1	0.00	1.19 ± 0.57
17 : 0	0.00	0.55 ± 0.28
18 : 1	45.69 ± 3.32	42.73 ± 4.32
18 : 0	4.53 ± 0.55	7.35 ± 1.51
18 : 2	3.77 ± 0.58	6.86 ± 2.57
20 : 1	0.94 ± 0.19	1.73 ± 0.39
20 : 0	0.00	0.02 ± 0.05

FAME: fatty acid methyl ester.
